# Prognostic Impact of Time to Ipsilateral Breast Tumor Recurrence after Breast Conserving Surgery

**DOI:** 10.1371/journal.pone.0159888

**Published:** 2016-08-05

**Authors:** Marie Gosset, Anne-Sophie Hamy, Peter Mallon, Myriam Delomenie, Delphine Mouttet, Jean-Yves Pierga, Marick Lae, Alain Fourquet, Roman Rouzier, Fabien Reyal, Jean-Guillaume Feron

**Affiliations:** 1 Department of Surgery, Institut Curie, 75005, Paris, France; 2 Breast Unit, Belfast City Hospital, Lisburn Road, Belfast, BT9 7AB, Northern Ireland; 3 Department of Medical Oncology, 75005, Institut Curie, Paris, France; 4 Paris Descartes University, 75006, Paris, France; 5 Department of Tumor Biology, Institut Curie, 75005, Paris, France; 6 Department of Radiotherapy, Institut Curie, 75005, Paris, France; 7 Residual Tumor and Response to Treatment Lab, Translational Research Department, Institut Curie, 75005, Paris, France; 8 UMR932 Immunity and Cancer, INSERM, Institut Curie, 75005, Paris, France; Fu Jen Catholic University, TAIWAN

## Abstract

**Background:**

The poor prognosis of patients who experience ipsilateral breast tumor recurrence (IBTR) after breast conserving surgery (BCS) is established. A short time between primary cancer and IBTR is a prognostic factor but no clinically relevant threshold was determined. Classification of IBTR may help tailor treatment strategies.

**Purpose:**

We determined a specific time frame, which differentiates IBTR into early and late recurrence, and identified prognostic factors for patients with IBTR at time of the recurrence.

**Methods:**

We analyzed 2209 patients with IBTR after BCS. We applied the optimal cut-points method for survival data to determine the cut-off times to IBTR. A subgroup analysis was performed by hormone receptor (HR) status. Survival analyses were performed using a Cox proportional hazard model to determine clinical features associated with distant-disease-free survival (DDFS) after IBTR. We therefor built decision trees.

**Results:**

On the 828 metastatic events observed, the majority occurred within the first 3 months after IBTR: 157 in the HR positive group, 98 in the HR negative group. We found different prognostic times to IBTR: 49 months in the HR positive group, 33 in the HR negative group. After multivariate analysis, time to IBTR was the first discriminant prognostic factor in both groups (HR 0.65 CI95% [0.54–0.79] and 0.42 [0.30–0.57] respectively). The other following variables were significantly correlated with the DDFS: the initial number of positive lymph nodes for both groups, the initial tumor size and grade for HR positive tumors.

**Conclusion:**

A short interval time to IBTR is the strongest factor of poor prognosis and reflects occult distant disease. It would appear that prognosis after IBTR depends more on clinical and histological parameters than on surgical treatment. A prospective trial in a low-risk group of patients to validate the safety of salvage BCS instead of mastectomy in IBTR is needed.

## Introduction

The oncological outcome (overall survival, distant metastasis, contralateral breast cancer rates) of breast-conserving surgery (BCS) combined with adjuvant radiotherapy is equivalent to mastectomy in the treatment of the early-stage breast cancer [1–5]. The incidence of ipsilateral breast tumor recurrence (IBTR) following BCS is estimated from 5 to 10% in studies with 10 years follow up from the initial tumor treatment [3,4,6–8].

Accurate estimation of prognosis for patients with IBTR is difficult due to complex interactions of multiple clinico-pathological features. Several authors have suggested that long-term prognosis remains good in recurrent lesions that are less than 2 cm, hormone receptor positive and absent nodal metastases [6,7,9–13]. Time from initial breast cancer treatment to IBTR was identified in several studies as a major prognostic factor. A short interval time is a strong determinant of risk for distant metastasis [6,7,10,14,15]. There is, however, no defined cut-off period to accurately categorize IBTR into early relapse and late relapse. Early and late IBTR may correspond to 2 distinct types of IBTR [11–13,16–18]: a) true recurrences, corresponding to re-growth of resistant cells after initial treatment, and b) new primary tumors, corresponding to *de novo* cancer (early relapse and late relapse respectively). Accurate classification of IBTR into early and late relapse may help better predict subsequent prognosis, as new primary tumors are considered to have an improved survival compared to true relapses [11–13,16–18].

In addition to increase prognostic accuracy, classification of IBTR may help tailor surgical treatment strategies. Current guidelines for the surgical management of all IBTR cases recommend performing a modified radical mastectomy [19,20]. In theory, however, surgical management of a new primary tumor could theoretically involve a further BCS procedure, provided that local condition would ensure an acceptable aesthetic result. Several retrospective studies have reported outcomes following “salvage” BCS for IBTR. Overall, the risk of second relapse was high (30%) but the five-year overall survival rate reached 80–90% [10,21–27]. These studies however identified a subset of cases with favorable characteristics (longer time to first relapse, smaller tumor size, node negativity) associated with a lower rate of further local relapse.

The aims of this study were to 1) determine a specific time frame, which differentiates IBTR into early relapse and late relapse. 2) identify prognostic factors for patients with IBTR at the time of the recurrence 3) Suggest options for the surgical management of early and late IBTR.

## Material and Methods

### Patients

All experiments were performed retrospectively and in accordance with the French Bioethics Law 2004–800, the French National Institute of Cancer (INCa) Ethics Charter and after approval by the Institut Curie review board and ethics committee (Comité de Pilotage of the Groupe Sein). In the French legal context, our institutional review board waived the need for written informed consent from the participants. Moreover, women were informed of the research use of their tissues and did not declare any opposition for such researches. Data were analyzed anonymously. We identified in the Institut Curie prospective breast cancer database all patients with primary invasive breast cancer treated by breast conserving surgery from 1980 to 2010, and who experienced subsequent ipsilateral breast tumor recurrence (IBTR). Patients who were diagnosed with distant metastases within 3 months of IBTR were also included, whereas patients with metastases or lymph node recurrence diagnosed prior to IBTR were excluded. The following clinical and pathological variables of primary breast cancer were available for analysis: age, BMI, initial tumor size and histological grade, initial nodal and hormonal status and adjuvant treatment. Prognostic impact of a continuous variable is difficult to demonstrate so we used categorization of age, as most previous studies [6,9,10,14,15,21,23,28]. Estrogen receptor (ER) and progesterone receptor (PR) status were determined as follows. After rehydration and antigenic retrieval in citrate buffer (10mM, pH 6.1), the tissue sections were stained for ER (clone 6F11, Novocastra, Leica Biosystems, Newcastle, UK; 1/200) and PR (clone 1A6, Novocastra, 1/200). Revelation of staining was performed using the Vectastain Elite ABC peroxidase mouse IgG kit (Vector, Burlingame, CA, USA) and diaminobenzidine (Dako A/S, Glostrup, Denmark) as chromogen. Positive and negative controls were included in each slide run. Cases were considered positive for ER and PR according to the standardised guidelines using a cutoff of greater than or equal to 10% stained tumor nuclei. Hormone receptors (HR) positivity was defined by positivity of either ER or PR, and HR negativity was defined by the negativity of both ER and PR.

Following BCS for the primary cancer, patients had received external radiotherapy according to national and local guidelines [20]. Adjuvant chemotherapy and hormonal therapy were also administrated according to national guidelines [20].

At the time of recurrence, all the patients had a radical mastectomy and adjuvant treatment following discussion at multidisciplinary meeting. Hormone receptor (HR) status and histological characteristics of the recurrence were unknown. Patients were followed up by physical examination every 6 months and mammography with or without ultrasonography every year.

### Statistical analysis

Baseline characteristics were described as a number (%) for qualitative variables and median (interquartile range, IQ) for quantitative variables. Data was compared using Chi-square tests for categorical variables and Student's t-tests for continuous variables.

We assumed from the literature that the time from initial treatment to IBTR diagnosis had prognostic significance on survival. Due to the possibility of a non-linear relationship between time to IBTR and survival, we applied the optimal cut-points method for survival data as defined by Contal et al [29] (R package survMisc). This method determines the cut-off time point yielding the most significant prognostic differences between two groups. The cut-off time point chosen minimizes the *p*-value linking the prognostic factor to outcome. Because of well-described differences of time-to relapse according to HR status [6,21,23,30], a subgroup analysis was performed by hormonal status.

Post relapse survival analyses were performed with the Kaplan-Meier estimate of the survival function. Primary endpoint was distant-disease-free survival (DDFS) after IBTR, defined as the time from first local relapse until distant disease. Survival curves were compared using log-rank tests. Estimation of hazard ratios and their associated 95% confidence interval was carried out using the Cox proportional hazard model. Age, initial adjuvant treatment, initial tumor size and histological grade, initial nodal and time to IBTR were included in the univariate analysis. Variables with a *p*-value of the log rank score test below 0.15 in univariate analysis were included in the multivariate model. Data were censored at 10 years. DDFS of each subgroup were estimated by the Kaplan–Meier method, they were compared using the log-rank test and plotted accordingly. Significance level was 0.05. A decision tree was established to identify homogeneous subgroups of patients and have a better clinical representation of the model. The rules of the construction of the decision tree are: the log-rank test p-value has to be significant and each subgroup defined by the discrimination has to include at least ten patients. Between several factors we choose the factor which minimizes the log-rank test p-value. Survival and metastasis free survival rates of the subgroups identified by the decision tree were estimated by the Kaplan–Meier method, and were compared using the log-rank test. All these estimations were then plotted. Significance level was 0.05. Analyses were performed using R software, 2.13.2 version (http://cran.rproject.org) using the following packages: glm, survival and rpart.

## Results and Discussion

### Results

A total of 5837 patients treated at Institut Curie had an IBTR after an initial invasive breast cancer locally treated by breast conserving surgery plus radiotherapy. We excluded 2641 patients diagnosed with distant metastases or lymph node metastases before the diagnostic of local recurrence, and 987 patients with unknown initial hormonal receptor (HR) status. Overall, 2209 patients remained for the analysis. The patients and the tumor characteristics of the primary tumor are summarized in Table 1, and are also described by hormone receptor (HR) status. Median age at cancer diagnosis was 49 years. The median tumor size was 2.0 cm, 67.8% had no lymph node involvement (n = 1291). Primary tumors were mostly HR positive (78.7%, n = 1738). HR negative tumors had a significantly larger tumor size (p<0.01), higher histological Elston and Ellis grade (p<0.001) and higher rates of lymph nodes involvement (p<0.01) at time of the initial diagnosis. The median interval time to local recurrence in the whole population was 68 months and was significantly longer in the HR-positive subgroup (78 months), when compared to the HR-negative subgroup (38 months) (p<0.01). After IBTR, the median follow-up after IBTR diagnostic was 54 months.

**Table 1:** Initial characteristics of the population with ipsilateral Breast Cancer Recurrence, at time of primary cancer.

Factors	Global population n = 2209	Hormone receptor Positive Tumors n = 1738	Hormone receptor Negative Tumors n = 471	p
Age (years)				
<40	389 (17.6%)	293 (16.9%)	96 (20.4%)	0.18
40–70	1723 (78.0%)	1366 (78.6%)	357 (75.8%)	
>70	97 (4.4%)	79 (4.5%)	18 (3.8%)	
BMI > 25	302 (23.6%)	244 (23.7%)	58 (23.3%)	0.51
Initial tumor size (cm)	2.0 (1.5–3.0)	**2.0 (1.5–3.0)**	**2.5 (2.0–4.0)**	**<0.01**
**No of positive nodes at time of the primary cancer**				
0 (pN0)	1291 (67.8%)	**1051 (70.1%)**	**240 (59.3%)**	**< 0.01**
1 or 3 (pN1)	441 (23.2%)	**335 (22.3%)**	**106 (26.2%)**	
> 3 (pN2)	172 (9.0%)	**113 (7.5%)**	**59 (14.6%)**	
*Unknown*	*305*	*239*	*66*	
**Initial tumor grade (Elston Ellis classification)**				
I	501 (23.8%)	**470 (28.2%)**	**31 (7.1%)**	**< 0.01**
II	804 (38.3%)	**718 (43.1%)**	**86 (19.8%)**	
III	796 (37.9%)	**478 (28.57%)**	**318 (73.1%)**	
*Unknown*	*108*	*72*	*36*	
**Adjuvant treatment**				
Radiotherapy	2177 (98.5%)	1713 (98.5%)	464 (98.5%)	0.99
Chemotherapy	832 (37.6%)	**555 (31.9%)**	**277 (58.8%)**	**< 0.01 **
Endocrine therapy	895 (40.5%)	**810 (46.6%)**	**85 (18.0%)**	**<0.01**
**Time to local recurrence (months)**				
	68 (35–124)	**78 (42–132)**	**38 (22–86)**	**< 0.01**
**Follow-up after local recurrence (months)**				
	54 (22–107)	**59 (25–113) **	**38 (17.5–81.5)**	**< 0.01**
**DDFS after local recurrence (months)**				
	37 (11–89)	**42 (14–93)**	**23 (4–68.5)**	**< 0.01**

**Fig 1:**
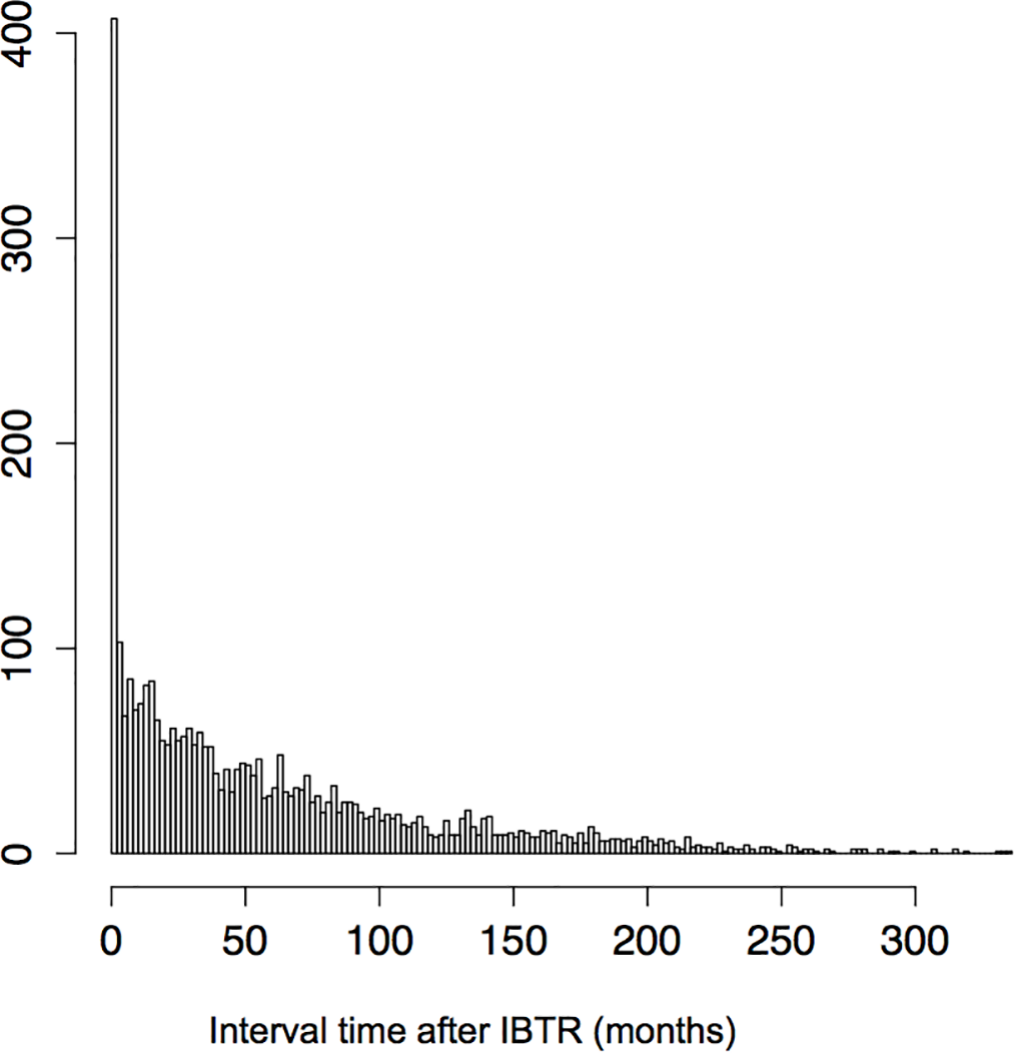
Distant disease occurrence according to time after IBTR for overall population. Vertical bars represent the number of events. Width of vertical bars stands for 3 months.

On 2209 patients with IBTR, 828 experience distant metastases (597 in HR positive group and 231 in HR negative group). The median time from IBTR to distant disease was 37 months, and was significantly different according to HR status (42 months in the HR-positive subgroup *versus* 23 months in the HR-negative subgroup, p<0.01). Distant Disease Free survival after IBTR was 67% at five years (95% CI 0.65–0.69). The wide majority of distant metastasis occurred early after IBTR with a peak in the incidence within the first 3 months after IBTR. We observed 157 metastatic events in the HR positive group and 98 in the HR negative group. (Fig 1, S1A and S1B Fig).

#### Timing of IBTR as predictor of DDFS

**Fig 2:**
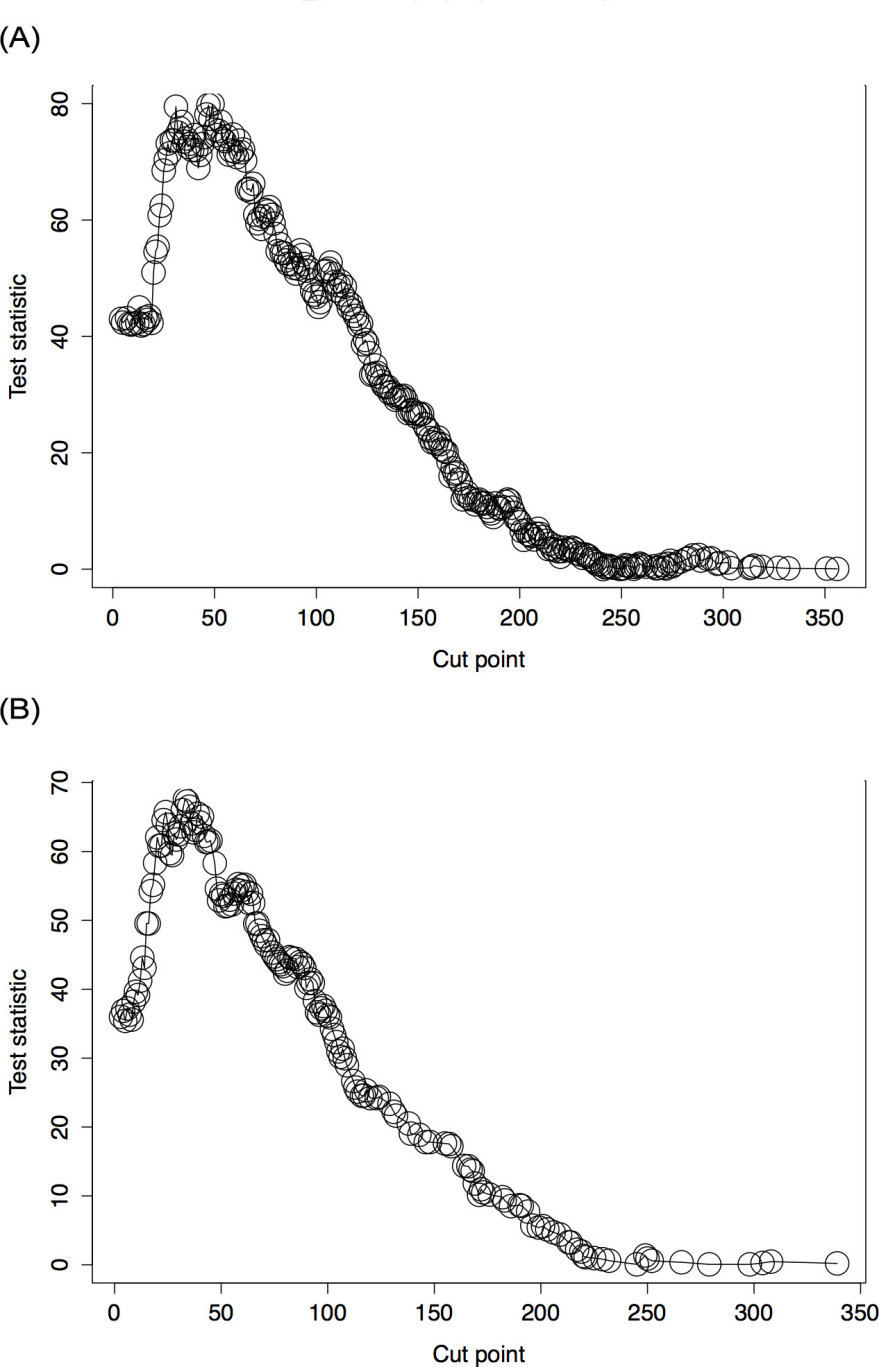
Cutpoint determination. (larger values indicate cut point more likely here). (A) Patients with hormone receptor positive tumors: 49 months. (B) Patients with hormone receptor negative tumors: 33 months.

The delay from initial breast cancer diagnostic to IBTR significantly correlated with prognosis (Distant Disease Free Survival). We identified 34 months as the cut-off for time to IBTR minimizing the *p*-value linking the time to IBTR to DDFS. This cut-off was different according to HR status (HR positive: 49 months versus HR negative: 33 months (Fig 2A and 2B).

#### Prognostic factors of post relapse DDFS

**Fig 3:**
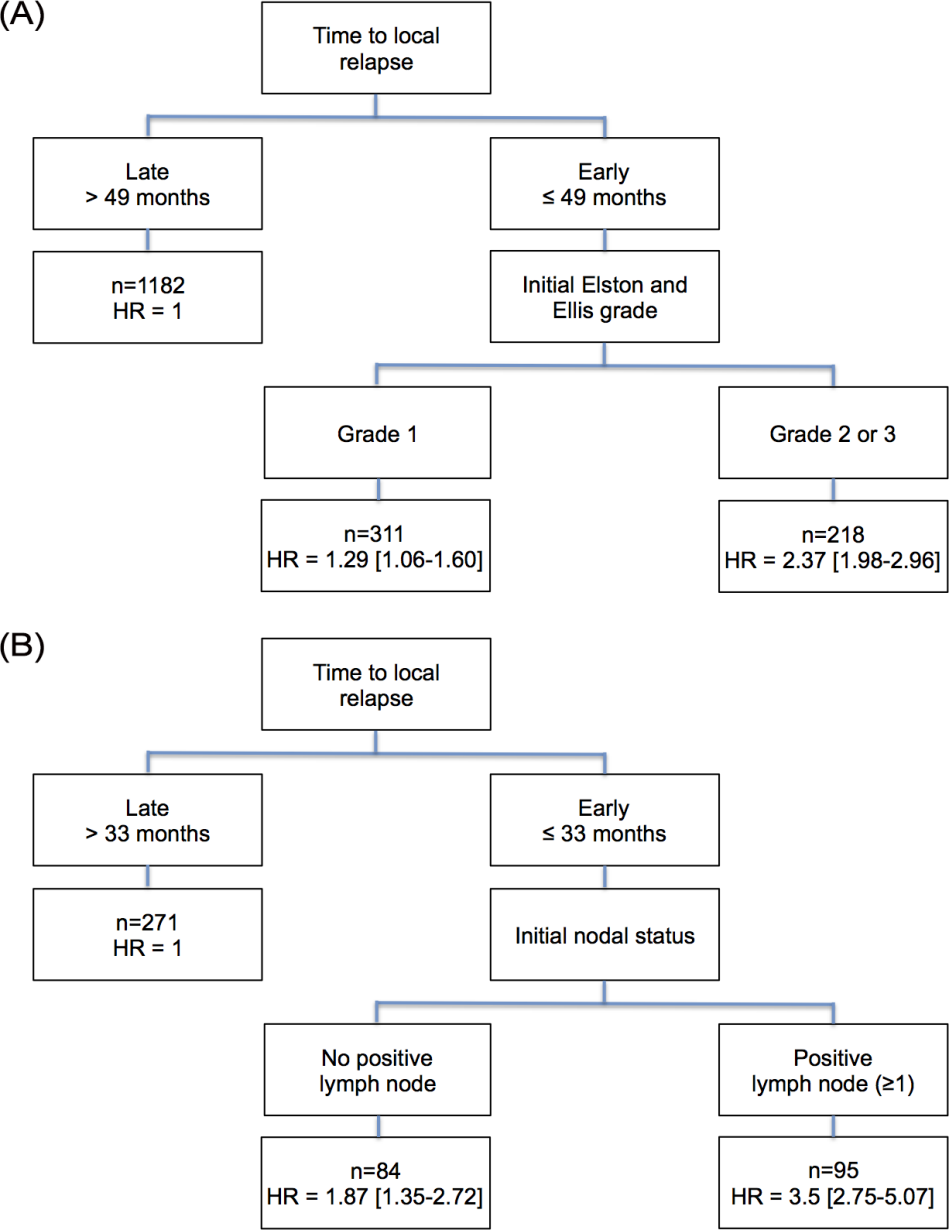
Decision tree algorithm HR = hazard ratio for distant disease at 10 years. (A) Patients with hormone receptor positive tumors. (B) Patients with hormone receptor negative tumors.

**Fig 4:**
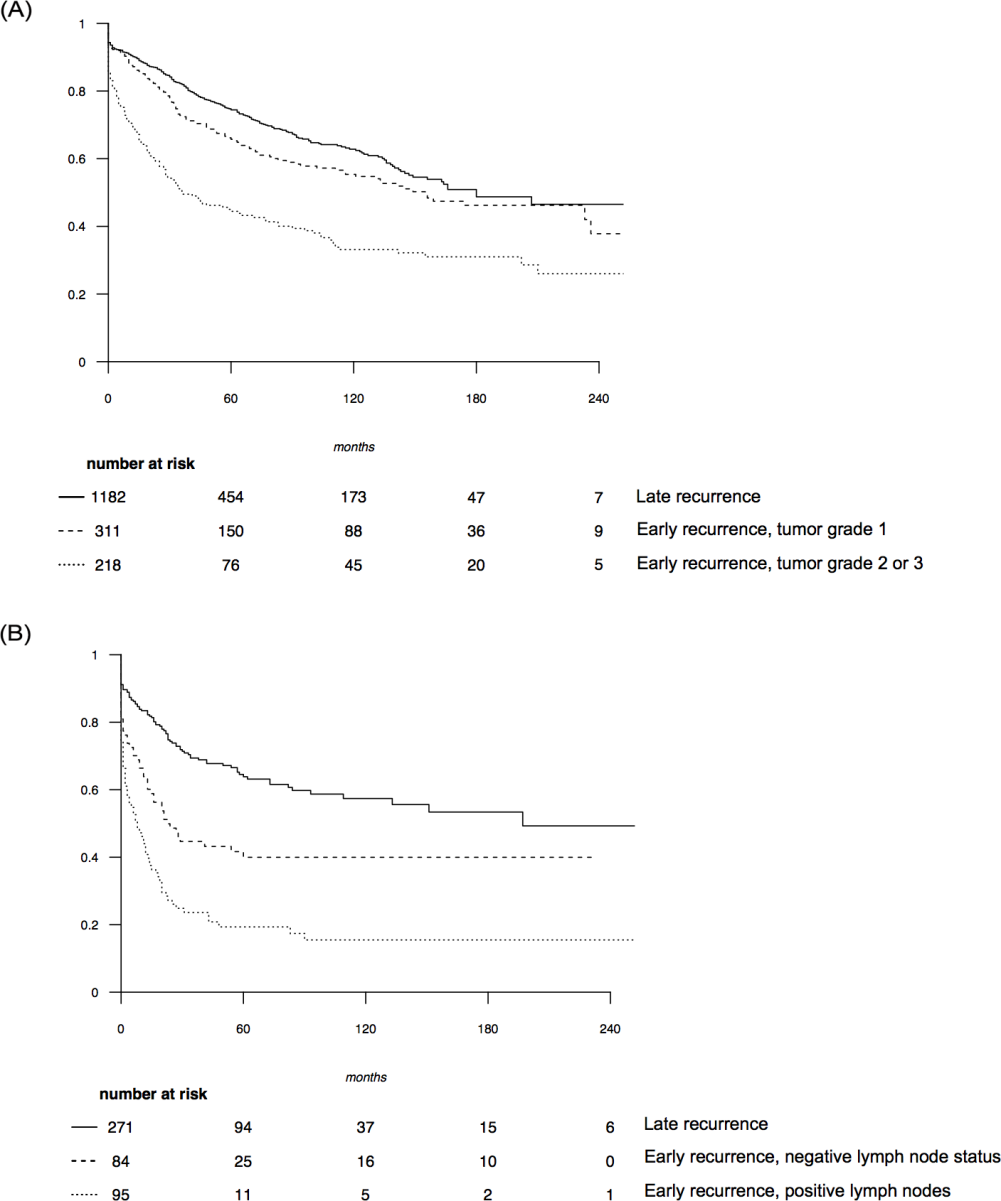
Distant disease free survival curves according prognostic subgroup. (A) Patients with hormone receptor positive tumors. (B) Patients with hormone receptor negative tumors.

After univariate analysis, the following variables were significantly associated with the DDFS for patients with HR positive initial breast cancer: age, initial adjuvant treatment, initial tumor size and grade, number of positive lymph nodes and time to IBTR (Table 2). After multivariate analysis, the initial tumor size and grade, the number of positive lymph nodes and the time to IBTR (HR 0.65 CI95% [0.54–0.79]) remained significantly associated with DDFS (Table 2). After applying the regression partition algorithm, time to IBTR was the first discriminant prognostic factor, then the initial tumor grade. Three prognostic subgroups were identified (Fig 3A): patients with late recurrence (> 49 months, reference group), patients with early recurrence (≤ 49 months) and initial tumor grade 1 (HR = 1.29 (CI95% [1.06–1.60]), and patients with early recurrence and initial tumor grade 2 or 3 (HR = 2.37 (CI95% [1.98–2.96]). The latter group had the poorest prognosis. The Kaplan-Meier curve of the distant disease-free survival for each subgroup is shown in Fig 4A.

**Table 2:** Prognostic factors for post relapse disease free survival–hormone receptor positive tumors.

Factors	Population	Event	Univariate analysis Hazard Ratio (CI 95%)	p	Multivariate analysis Hazard Ratio (CI 95%)	p
**Age at initial diagnosis (years)**						
< 40	293	136	1		1	
40–70	1366	438	0.72 [0.59–0.87]	< 0.001	0.8 [0.64–1]	0.05
>70	79	23	1.03 [0.66–1.61]	0.8	1.32 [0.78–2.25]	0.3
**Initial adjuvant treatment**						
Chemotherapy	271	113	1.54 [1.22–1.94]	< 0.001	1.04 [0.78–1.38]	0.8
Endocrine therapy	526	173	1.46 [1.19–1.80]	< 0.001	1.30 [1.01–1.67]	0.04
Chemo and endocrine therapy	284	117	2.13 [1.69–2.68]	< 0.001	1.24 [0.92–1.67]	0.038
**Tumor size at initial diagnosis**						
< or = 2cm	557	139	1		1	
2–5cm	1023	413	1.57 [1.3–1.91]	< 0.001	1.27 [1.03–1.57]	0.02
> 5cm	39	19	2.2 [1.36–3.56]	0.0001	1.37 [0.8–2.35]	0.3
**Initial tumor grade**						
1	470	106	1			
2	718	236	1.50 [1.19–1.88]	< 0.001	1.43 [1.11–1.87]	0.008
3	478	231	2.28 [1.81–2.87]	< 0.001	1.94 [1.48–2.56]	< 0.001
**Positive nodes at initial diagnosis**						
0	1051	306	1			
1 to 3	335	133	1.58 [1.29–1.94]	< 0.001	1.39 [1.10–1.76]	0.006
> 3	113	71	3.34 [2.58–4.33]	< 0.001	2.33 [1.72–3.17]	<0.001
**Time to IBTR (< or = 49 months versus > 49 months)**						
Early	557	279	1		1	
Late	1182	318	0.59 [0.5–0.69]	<0.001	0.65 [0.54–0.79]	<0.001

In HR negative tumors, after univariate analysis, the following variables were significantly correlated with the DDFS: initial adjuvant chemotherapy, initial tumor size and grade, number of positive lymph nodes and time to IBTR. In multivariate analysis, the initial number of positive lymph nodes and the time to IBTR remained significant (Table 3). As for positive HR tumors, time-to-IBTR was the first discriminant prognostic factor after applying the recursive partition tree algorithm, and then the initial lymph node status. Three prognostic subgroups were also identified (Fig 3B): patients with late recurrence (> 33 months, reference group), patients with early recurrence (≤ 33 months) and initial negative lymph node status (HR = 1.87 (CI95% [1.35–2.72]), and patients with early recurrence and positive lymph nodes (HR = 3.5 (CI95% [2.75–5.07]). The latter group had the poorest prognosis. The Kaplan-Meier plot of DDFS for each subgroup is presented in Fig 4B.

**Table 3:** Prognostic factors for post relapse disease free survival–hormone receptor negative tumors.

Factors	Population	Event	Univariate analysis Hazard Ratio (CI 95%)	p	Multivariate analysis Hazard Ratio (CI 95%)	p
**Age at initial diagnosis (years)**						
< 40	96	45	1			
40–70	357	179	1.15 [0.83–1.59]	0.4		
>70	18	7	1.06 [0.48–2.36]	0.9		
**Initial adjuvant treatment**						
Chemotherapy	240	127	1.79 [1.3–2.46]	< 0.001	1.14 [0.74–1.76]	0.6
Endocrine therapy	48	23	1.73 [1.09–2.75]	0.02	1.47 [0.83–2.6]	0.2
Chemo and endocrine therapy	37	27	2.24 [1.38–3.65]	0.001	1.25 [0.66–2.37]	0.5
**Tumor size at initial diagnosis**						
< or = 2cm	99	32	1		1	
2–5cm	310	168	1.91 [1.31–2.78]	< 0.001	1.39 [0.92–2.09]	0.1
> 5cm	37	21	2.63 [1.52–4.57]	< 0.001	1.43 [0.78–2.61]	0.2
**Initial tumor grade**						
1	31	9	1			
2	86	37	1.61 [0.78–3.35]	0.2	1.24 [0.47–3.27]	0.7
3	318	173	2.16 [1.11–4.23]	0.02	1.55 [0.62–3.88]	0.3
**Positive nodes at initial diagnosis**						
0	240	96	1			
1 to 3	106	62	1.61 [1.17–2.21]	0.004	1.39 [0.96–2.01]	0.09
> 3	59	45	2.98 [2.09–4.27]	< 0.001	2.23 [1.49–3.34]	<0.001
**Time to IBTR (< or = 33 months versus > 33 months)**						
Early	200	137	1		1	
Late	271	94	0.37 [0.29–0.48]	<0.001	0.42 [0.30–0.57]	<0.001

## Discussion

Using one of the largest datasets of patients with IBTR published to date, we confirmed the poor prognosis of patients with IBTR, since 37% of the patients developed metastasis and the majority occurred early after IBTR. We identified time from initial cancer to IBTR as a major prognostic factor. In addition to previous studies we have defined the interval time to local recurrence, which has the strongest significant prognostic factor for DDFS after an IBTR. We identified a delay to IBTR threshold with different cut-offs according to HR status (49 months for hormone positive tumors, and 33 months for hormone negative tumors). We subsequently developed simple decision trees to identify distinct prognostic subgroups, and time to IBTR remained the most important prognostic factor in both groups. Other prognostic factors identified differed according to HR status. The second discriminant prognostic factor was the initial tumor grade for HR positive tumors, but the initial lymph node status for HR negative tumors. The initial characteristics of the two populations were different: patients with HR negative tumors had higher initial tumor grade (p<0.01) and a lymph node involvement more frequent (p<0.01). As most of these patients had a high initial tumor grade (73.1% of grade 3), the initial tumor grade didn’t remain a significant prognostic factor in the multivariate analysis for HR negative tumors. In contrast, most of patients with HR positive tumors had no lymph node involvement (70.1%) and this factor was consequently not found as the most discriminant.

The poor prognosis of patients who experience IBTR is well established [4,6,9,31,32]. About 10% of patients are diagnosed with distant disease at time of local relapse, before any local treatment [9,31,33,34]. In our study, more than a third of the patients developed metastasis and the majority of metastasis occurred early, within 3 months IBTR diagnosis. As we know, metastases diagnosed within 3 months of cancer are usually considered as synchronous metastases. This main result shows that isolated breast cancer recurrence is associated with undetected distant disease. But clinical situations are heterogeneous and accurate identification of high-risk patients is difficult. We found that time to IBTR is the most important factor to discriminate prognostic subgroups.

There is evidence that time intervals between the treatment of primary breast cancer and IBTR affect survival [14,15]. As confirmed by our findings, a short time interval is a strong determinant of poor prognosis [6,9,10,23,35–37]. Many empirical interval time cut-off values were reported in the literature (one, 2 or 5 years) [6,9,10,14,23,35–37], but no single threshold clinically relevant was determined. The originality of our work was to use the cut-points method, which allows good discrimination between prognostic subgroups. Furthermore, to our knowledge, no author took into account the HR status, while natural history and prognosis of positive and negative HR tumors are inherently different [6,21,23]. The magnitude of the prognostic impact of time to IBTR was higher in the HR-negative group (Hazard ratio: 0.65 [0.54–0.79]) than in the HR-positive tumors (0.42[0.30–0.57)). This is in line with the shape of the survival curves: for negative HR tumors, a clear breakpoint in the survival curves is obvious, while the slope of the curve in the positive HR tumors is more linear.

Such a prognostic impact of interval time to IBTR is in agreement with the concepts of true relapse versus new primitive tumor. It has been shown that patients with a new primary cancer have a better prognosis that those with a true relapse [10–13]. Early recurrences may indicate primary chemoresistant or radiotherapy-resistant tumors. In these cases, local recurrence refers to a true relapse related to resistant residual tumor cells and represents a high-risk subgroup of occult metastatic disease. In contrast, later recurrences may be more likely new primary tumors [11]. These “second primary cancers” represent a localized disease. Several studies have attempted to classify these two distinct types [11,17,38–40]. Most of the classifications are based on the location of the relapse and the histologic subtype. Huang et al classified relapse as true recurrence if it appears within 3cm of the primary cancer and is the same histology [11]. Others publications focused on the clonal stability between the primary cancer and the recurrence. For example, Smith et al. classified tumors as new primitive if the flow cytometry changed from aneuploid to diploid [17]. Based on allele imbalance or loss of heterozygosity, Schlechter et al. investigated whether quantitative DNA fingerprinting could distinguish second primary cancer from true recurrence [39]. However, these classifications based on cancer gene signature [41–45] are still debated and there is a lack of relevant signatures to predict the response to chemotherapy and targeted treatment [46,47]. Oncologists still continue to primarily rely on clinical and pathological characteristics to make decisions.

The main limit of this study is the lack of histologic and therapeutic knowledge of the recurrence. To assess prognostic impact of time to IBTR, systemic therapy after IBTR may be another important factor. All patients had a salvage mastectomy, but data are not available concerning chemotherapy. In addition, we should evaluate the decision tree algorithm to distinguish true recurrence from new primary. But no clinical and pathological characteristics were available concerning the local recurrence except the time of diagnosis. Nevertheless, our data suggests that the time interval between the treatment of primary cancer and IBTR could help to discriminate between true relapse and new primary cancer.

There is no standard management for patients who experience IBTR. Given the poor prognoses of these patients [4,6,9,31,32], new therapeutic strategies must be considered.

Concerning systemic treatment, the benefit of a second chemotherapy has been proven in one randomized trial [32,48,49]: CALOR is a randomized trial comparing adjuvant chemotherapy to simple monitoring for IBTR. The 5 years disease free survival was 69% with chemotherapy versus 57% without chemotherapy (HR 0.59 p = 0.046) [49]. The authors concluded that a second line adjuvant chemotherapy should be recommended for IBTR, because occult metastatic disease is present. But the subgroup analysis did not show any effect of chemotherapy in the HR positive group. There is no solid data concerning endocrine therapy. One randomized trial (SAKK23/82) compared tamoxifen versus control for “good prognosis” local relapse after mastectomy [50]. It did not show any difference in overall survival with a median follow-up time of 11.6 years. But data lead to believe that the use of adjuvant hormonetherapy in patients with HR-positive IBTR is justified. Patients who experienced recurrence on tamoxifen should be offered treatment with an aromatase inhibitor while patients who have progressed on an aromatase inhibitor may be reconsidered for tamoxifen [51].

Concerning locoregional treatment, the impact on metastatic risk is not demonstrated. Local recurrence is a strong independent predictor factor of distant disease and poor survival [35,52]. Currently, mastectomy is still considered the treatment of choice for IBTR [19,20] because of a high rate (30%) of second local relapse [10,21–25] but impact on distant disease remains unclear. The surgical margin is one of the most important risk factor of local recurrence [53–55]. However, especially thanks to oncoplastic technics, BCS allows large resections and free margin as total mastectomy. Total mastectomy with reconstruction is a therapeutic strategy increasingly used [56,57]. Skin sparing and nipple sparing mastectomies allow good cosmetic outcomes [58,59], but postoperative complications such as infections, necrosis exist. Plus, psychological impact of mastectomy is important, even after immediate reconstruction, and skin or nipple sparing cannot always be guaranteed [60]. Patients treated with BCS for primary cancer have a better body image [61] and there is no psychological or cosmetic advantage of one type of reconstruction over another. Surgery must be adapted to the patient morphology and psychological experience. We believe that second BCS represents a relevant option in 2016. The use of a second BCS was first described in the retrospective study of Kurtz et al. in 1991 [62] with no significant difference in 10 year OS between second BCS and mastectomy. Further conservative procedure was associated with 23% of second local relapse. Thereafter, several authors showed that for patients diagnosed with a local recurrence of less than 2 cm, hormone receptor positive and no lymph node involvement, the long-term prognostic remain good [6,7,9–13]. In such low-risk groups, a high rate of five-year overall survival is observed, between 80 and 90% [10,21–25]. In the retrospective study of Chen et Martinez [21] on 747 patients with IBTR, 179 patients experienced a second lumpectomy and 569 a mastectomy. After univariate analysis, patients undergoing second BCS had worse overall survival (with five-year survival of 67% versus 78% for the BCS and mastectomy groups respectively), but a subgroup analysis in HR positive tumors showed an opposite trend. For patients with a HR positive tumor less than 2cm, without lymph node involvement, the overall survival was 62% after lumpectomy and 57% after salvage mastectomy (p = 0.09). Likewise, the meta-analysis of Vila et al. [24] suggested that patients older than 50 years with a small unifocal recurrence (< 2cm) and time to IBTR longer than 48 months could undergo a second BCS. More recently, Lee et al. confirmed that clinical and histological criteria may be more important than the type of surgery regarding prognosis after IBTR [15]. These results are difficult to interprete because these retrospective studies included a small number of patients. Thus, neither selection bias nor lack of statistical power could be excluded. Indeed, patients treated with a second BCS are more likely to have smaller tumors with better prognostic factors than patients who underwent mastectomy. But all together, these data suggest that metastatic risk may be independent of the local treatment. Concerning radiotherapy, no guidelines exist. New emerging radiation techniques could provide effective local control while limiting toxicity of a repeat breast irradiation. Several strategies have been studied such as partial breast irradiation [63] or brachytherapy [64–66]. The RTOG (Radiation Therapy Oncology Group) phase II study of repeat breast preserving surgery and 3D-conformal partial breast re-irradiation is now closed to accrual [67]. Results are pending.

## Conclusions

To conclude, a short interval time to IBTR is the strongest factor of poor prognosis. Early isolated breast cancer recurrence is a marker of undetected distant disease. Our decision trees suggest that only few criterions–time between primary cancer and IBTR, hormone status and tumor grade of the primary tumor, initial lymph nodes status–allow to discriminate prognostic subgroups. New therapeutic strategies for IBTR must be considered. Patients with late recurrence and good prognostic factors should be considered as having a local disease. In this group, second breast conserving surgery must be considered. The risk of a second local recurrence is then high, but close to the one after an initial BCS without radiotherapy [3,4]. Based on the literature and our results, it would appear that prognosis of patients after IBTR depends more on clinical and initial histological parameters than on surgical treatment. A prospective trial in a low-risk group of patients to validate the safety of salvage BCS instead of mastectomy in IBTR is therefore needed.

## Supporting Information

S1 Fig Distant disease occurrence according to time after IBTR for each hormone subgroup. Vertical bars represent the number of events. Width of vertical bars stands for 3 months. (A) Patients with hormone receptor positive tumors. (B) Patients with hormone receptor negative tumors.
(PDF)
