# Supplementary material for: Prognostic Impact of Time to Ipsilateral Breast Tumor Recurrence after Breast Conserving Surgery
Source: PLoS One. 2016 Aug 5;11(8):e0159888. doi: 10.1371/journal.pone.0159888 (PMC4975471; doi:10.1371/journal.pone.0159888)
Supplement: S1 Fig — Vertical bars represent the number of events. Width of vertical bars stands for 3 months. (A) Patients with hormone receptor positive tumors. (B) Patients with hormone receptor negative tumors. (PDF) [file pone.0159888.s001.pdf]

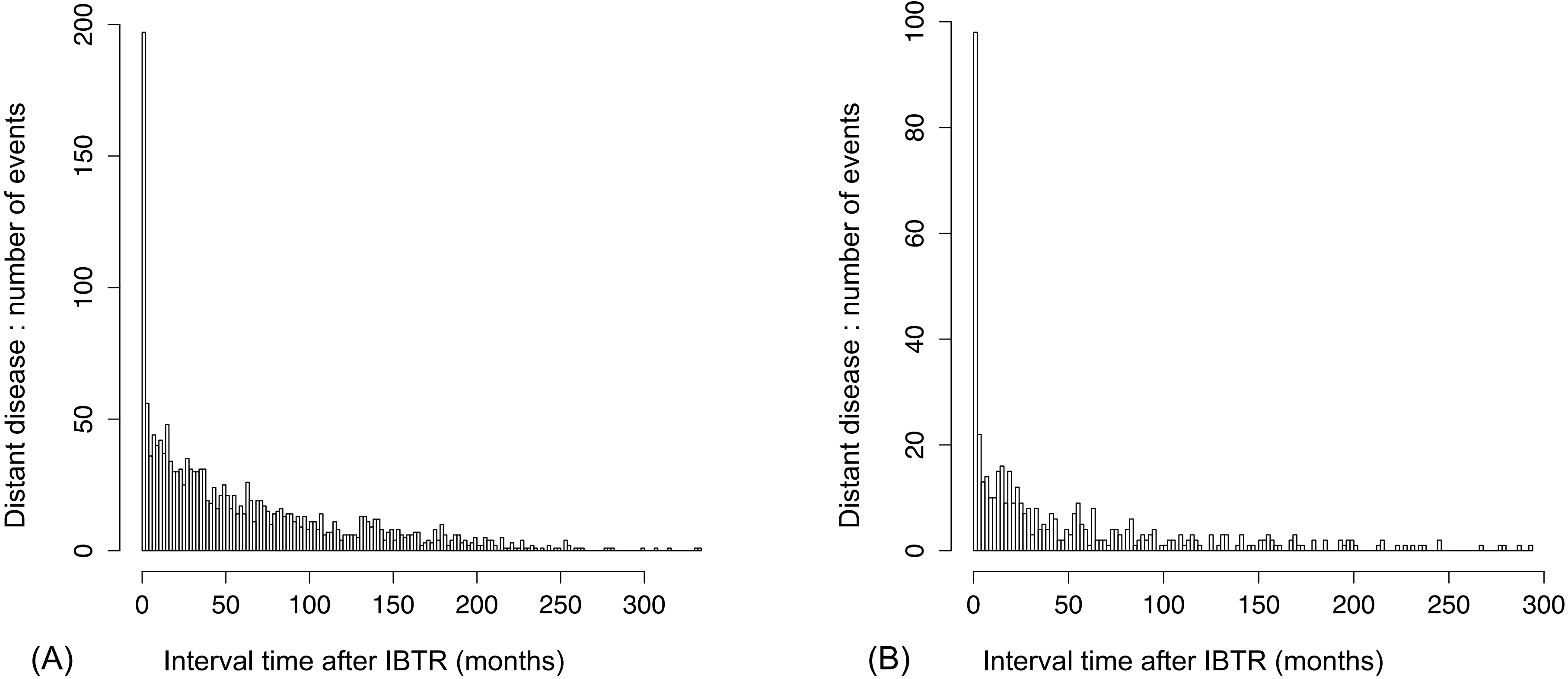

Distant disease : number of events
0
50
100
150
200
0
50
100
150
200
250
300
(A)
Interval time after IBTR (months)
Distant disease : number of events
0
20
40
60
80
100
0
50
100
150
200
250
300
(B)
Interval time after IBTR (months)
